# Adherence and persistence to tafamidis treatment among Medicare beneficiaries in the presence of a patient assistance program

**DOI:** 10.1038/s41598-024-62660-5

**Published:** 2024-07-15

**Authors:** Haechung Chung, Cera Cantu, Cindi Pankratova, Jason Kemner, Jose Alvir, Sapna Prasad, Yong Chen

**Affiliations:** 1 Pfizer Inc, New York, NY USA; 2 Clarify Health Solutions, New York, NY USA

**Keywords:** Health care economics, Outcomes research

## Abstract

Tafamidis is the only disease-modifying therapy approved to treat patients in the United States with transthyretin amyloid cardiomyopathy (ATTR-CM), which most commonly affects patients aged ≥ 65 years. The manufacturer operates a patient assistance program (PAP) to support access to tafamidis. This study conducted Privacy Preserving Record Linking (PPRL) using Datavant tokens to match patients across Medicare prescription drug plan (PDP) and PAP databases to evaluate the impact of PAPs on treatment exposure classification, adherence, and persistence determined using Medicare PDP data alone. We found 35% of Medicare PDP patients received tafamidis through the PAP only; 14% through both Medicare PDP and the PAP, and 51% through Medicare PDP only. Adherence and persistence were comparable between these cohorts but underestimated among patients who received ≥ 2 prescriptions through Medicare PDP and ≥ 1 through the PAP when solely using Medicare data versus pooled Medicare and PAP data (modified Medication Possession Ratio: 84% [69% ≥ 80% adherent] vs. 96% [93%]; Proportion of Days Covered: 77% [66% ≥ 80% adherent] vs. 88% [88%]; mean days to discontinuation: 186 vs. 252; total discontinuation: 13% vs. 11%). Cross-database PPRL is a valuable method to build more complete treatment journeys and reduce the risk of exposure misclassification in real-world analyses.

## Introduction

Administrative claims data are widely used to address research questions on the real-world use and effectiveness of clinical therapies^1,2^. In the United States, a high proportion of older people are Medicare beneficiaries, and the Medicare prescription drug plan (PDP) database is commonly used to evaluate treatment journeys in patients ≥ 65 years of age. However, patients may also obtain healthcare through other routes, such as private health insurance, self-funded healthcare, and patient assistance programs (PAPs). Healthcare obtained via these routes is not fully captured in the Medicare PDP database.

Privacy Preserving Record Linking (PPRL) using tokenization technology substitutes personal identifying information (PII) for de-identified ‘tokens’. These tokens act as keys and can facilitate faithful matching of patients across claims databases while preserving anonymity^3,4^. Though not yet widely used, PPRL can aid in the identification of both paid and unpaid treatment utilization, and has potential to improve the quality and scope of real-world claims data analyses^3,5–8^.

Transthyretin amyloid cardiomyopathy (ATTR-CM) is a progressive and often fatal condition, typically diagnosed in patients > 65 years^9,10^. It causes restrictive heart failure due to the accumulation of transthyretin (TTR) amyloid in the myocardium^9^. Until recently, there was no disease-modifying therapy for patients with ATTR-CM, and life expectancy was limited to ~ 2–6 years after diagnosis^9,11,12^. In 2019, tafamidis, a TTR stabilizer, became the first US Food and Drug Administration (FDA)-approved disease-modifying treatment for patients with ATTR-CM^11^. To support access to tafamidis for patients with low incomes, the manufacturer maintains an active PAP where the drug is provided at no cost. Many manufacturers operate similar PAPs to improve access to their innovative prescription therapies^13^.

Through the example of tafamidis, this analysis aimed to assess the impact of PAPs on exposure misclassification, treatment adherence and persistence calculations derived solely from a single traditional real-world data source. Specifically, we hypothesized that the addition of missing PAP data through PPRL would alter tafamidis treatment patterns observed among Medicare PDP beneficiaries.

## Results

### Privacy preserving record linking

**Figure 1:**
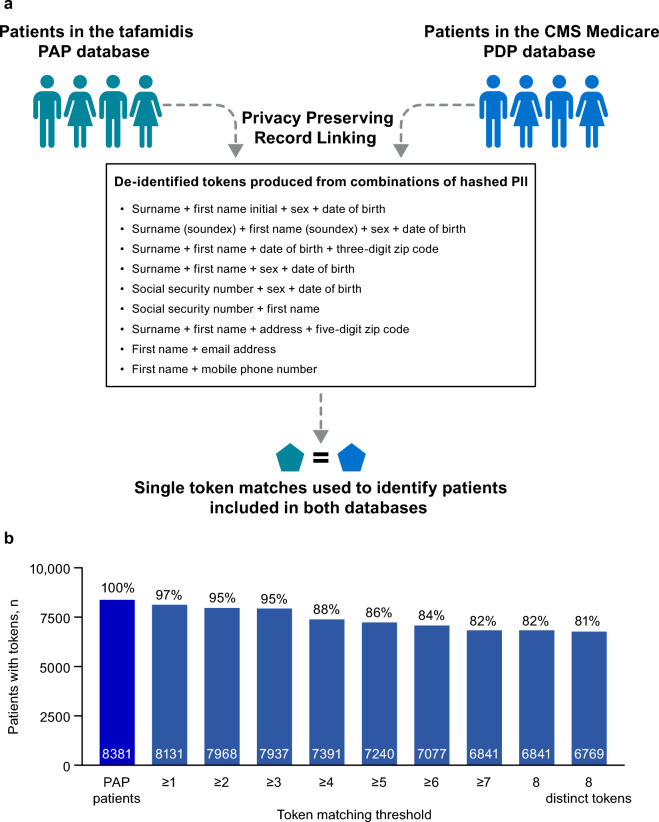
PPRL process and findings. CMS, Centers for Medicare & Medicaid Services; PAP, patient assistance program; PII, personally identifiable information; PDP, prescription drug plan; PPRL, Privacy Preserving Record Linking.

Privacy Preserving Record Linking using tokenization technology from Datavant, Inc.^8,9,11^ was used to match patients in the US Centers for Medicare & Medicaid Services (CMS) PDP and PAP databases with ≥ 1 tafamidis prescription at the approved dose (tafamidis meglumine 80 mg or tafamidis free acid 61 mg) between May 3, 2019, and December 31, 2021 (Fig. 1). The PDP database included drug claims from Medicare fee-for-service and Advantage beneficiaries. Of the 8381 patients in the PAP database with tokens, 8131 (97%) were matched to Medicare PDP beneficiaries with ≥ 1 token. The number of matched patients declined with increasing token thresholds, stabilizing at ≥ 7 tokens. To minimize the number of false positives, a threshold of 8 distinct tokens was used (i.e., only 1 patient from the PAP and 1 from the CMS PDP database had exactly the same set of tokens), resulting in 81% of patients in the PAP being matched in the Medicare PDP database.

### Cohorts by source of treatment

**Figure 2:**
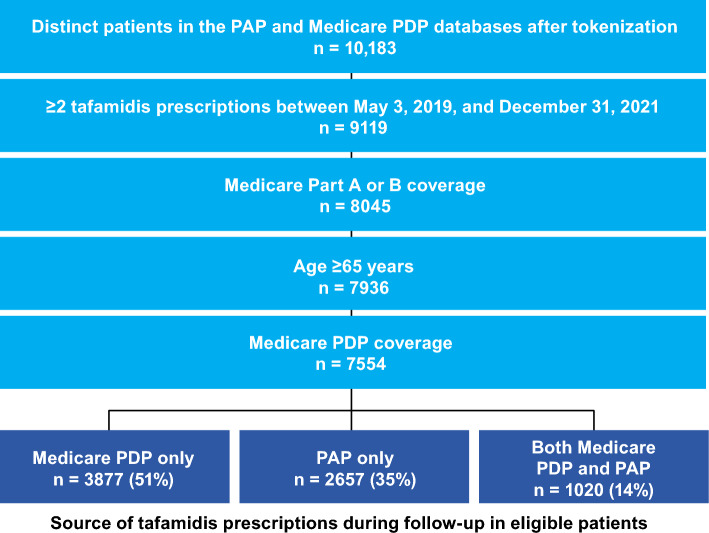
Patient attrition and cohorts. PAP, patient assistance program; PDP, prescription drug plan.

Among the 10,183 distinct patients matched in the PAP and Medicare PDP databases, 7554 met the inclusion criteria (age ≥ 65 years, Medicare FFS Parts A or B, and PDP coverage, and ≥ 2 tafamidis prescriptions on distinct dates, at the approved dose, from Medicare PDP or the PAP between May 3, 2019, and December 31, 2021). Of these 7554 patients, 3725 were enrolled in Medicare Advantage during the study period or in the 12 months prior to index (date of first prescription). Each of the 7554 patients were each included in one of the 3 cohorts (Fig. 2); half of the 7554 eligible patients received tafamidis through Medicare PDP only (n = 3877; 51%), approximately one-third received tafamidis through the PAP only (n = 2657; 35%); and the remaining patients received tafamidis through both Medicare PDP and the PAP (1020; 14%).

The demographics and enrollment characteristics of patient cohorts at index are shown in Table 1. The mean age among all eligible patients was 80 years, 81% were male, and 73% were White. Mean age, and the proportions in each sex, and race, were comparable among cohorts. The proportion of patients receiving their first prescriptions each year (index rate) was steady across the identification period in patients receiving tafamidis from Medicare PDP only, with one-third indexed in 2019 (33%), another third in 2020 (32%), and the final third in 2021 (35%). There was an increasing annual index rate among patients who only received tafamidis through the PAP, with 15% indexed in 2019, over a third indexed in 2020 (35%), and half (49%) in 2021. Index rate decreased over the identification period in patients who received tafamidis through Medicare PDP and the PAP, with nearly half (46%) indexed in 2019, one-third (32%) in 2020, and one-fifth (22%) in 2021. Perhaps reflecting the index rates over time, the longest follow-up times were observed in patients who received tafamidis through both Medicare PDP and the PAP (mean: 557 days), and shortest in patients who received tafamidis in the PAP only (mean: 332 days).

**Table 1:** Patient demographics and enrollment characteristics at index.

	Received tafamidis prescriptions during follow-up from			All patients n = 7554
	Medicare PDP only n = 3877	PAP only n = 2657	Both Medicare PDP and the PAP n = 1020	
Age, years				
Mean (SD)	80 (7)	80 (7)	79 (6)	80 (7)
Median (range)	80 (65–101)	80 (65–101)	79 (65–96)	80 (65–101)
Sex, n (%)				
Male	3135 (81)	2153 (81)	839 (82)	6127 (81)
Female	742 (19)	504 (19)	181 (18)	1427 (19)
Race, n (%)				
White	2678 (69)	2076 (78)	769 (75)	5523 (73)
Black	1004 (26)	508 (19)	210 (21)	1722 (23)
Hispanic	45 (1)	< 11	< 11	59 (1)
Asian	35 (1)	< 11	< 11	45 (1)
Other	50 (1)	28 (1)	13 (1)	91 (1)
Missing	65 (2)	31 (1)	18 (2)	114 (2)
Year of first prescription (index year), n (%)				
2019	1298 (33)	406 (15)	468 (46)	2172 (29)
2020	1238 (32)	938 (35)	331 (32)	2507 (33)
2021	1341 (35)	1313 (49)	221 (22)	2875 (38)
Follow-up time, days				
Mean (SD)	395 (279)	332 (240)	557 (259)	395 (272)
Median (range)	333 (5–952)	263 (1–927)	592 (1–931)	333 (1–952)

Low n values (< 11) were suppressed.

PAP, patient assistance program; PDP, prescription drug plan.

Cardiovascular, and other comorbidities commonly associated with ATTR-CM and observed in the 12 months prior to index are shown in Supplementary Table S1. The proportion of patients with chronic conditions included in the Chronic Conditions Data Warehouse listing are shown in Supplementary Table S2. Around half of all patients had comorbid cardiac arrhythmias, hypertension, or hyperlipidemia. The proportions of patients with each comorbid condition were comparable in each cohort.

Cardiovascular medications taken at index are summarized in Supplementary Table S3. The most common medications among all patients were diuretics (88%), beta-blockers (70%), and anticoagulants (61%). The proportions of patients taking each type of medication were closely aligned in each cohort.

### Adherence and persistence

**Figure 3:**
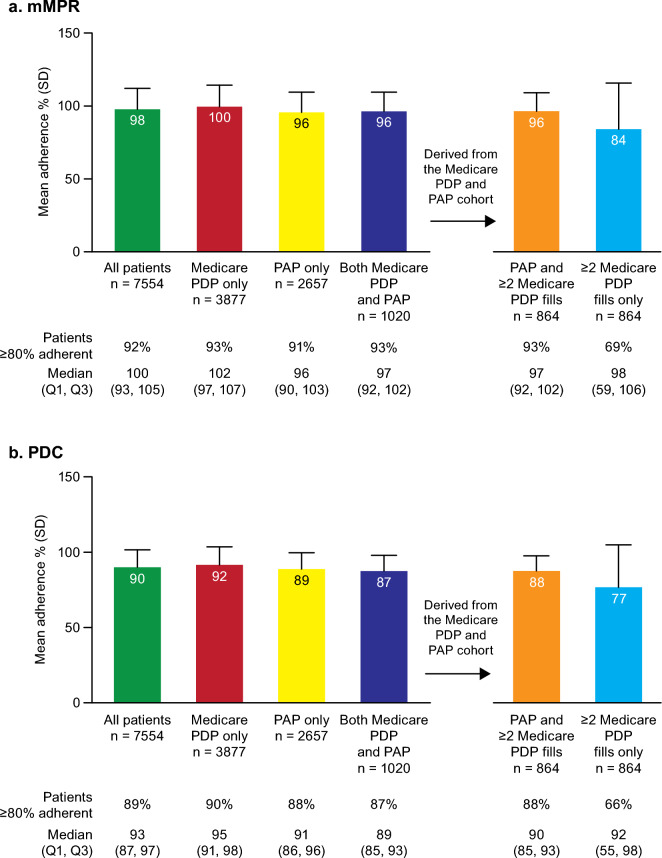
Adherence. mMPR, modified Medication Possession Ratio; PAP, patient assistance programs; PDC, proportion of days covered; PDP, prescription drug plan; Q, quartile; SD, standard deviation.

**Figure 4:**
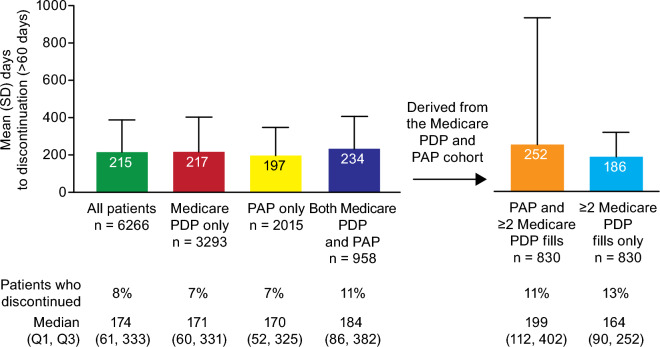
Persistence. PAP, patient assistance programs; PDP, prescription drug plan; Q, quartile; SD, standard deviation.

More than 86% of patients in the Medicare PDP only, PAP only, and Medicare PDP and PAP cohorts showed adherence in the modified Medication Possession Ratio (mMPR) and Proportion of Days Covered (PDC; Fig. 3). Persistence was broadly comparable among these cohorts, being longest in those who received tafamidis from both Medicare PDP and PAP (mean time to discontinuation: 234 days) and shortest in those who received through the PAP only (mean: 197 days; Fig. 4). A sensitivity analysis where a > 90 days gap was considered discontinuation (vs. a > 60 days gap) showed a similar trend (Supplemental Fig. 1).

To better evaluate the impact of the PAP on adherence and persistence measures calculated without database cross-linkage, we assessed outcomes in the Medicare PDP and PAP cohort using Medicare data only. The inclusion criteria for the study required patients to have ≥ 2 tafamidis prescriptions recorded on distinct service dates in either the Medicare PDP or PAP databases, but we found that 156 (15%) of patients in this cohort had only 1 prescription fill through Medicare PDP. To accurately calculate the outcomes, the additional analysis required ≥ 2 tafamidis prescription fills through Medicare PDP. Compared with findings from the overall Medicare PDP and PAP cohort, adherence was numerically similar in the subset of patients who had ≥ 2 tafamidis prescription fills through Medicare PDP (mMPR: 96% [93% were ≥ 80% adherent]; PDC: 88% [88% were ≥ 80% adherent]; Fig. 3), whereas persistence was slightly higher (mean days to discontinuation: 252; 11% discontinuation; Fig. 4). When utilizing only Medicare PDP data in this subgroup, we saw a decrease in adherence (mMPR: 84% [69% were ≥ 80% adherent]; PDC: 77% [66% were ≥ 80% adherent]), and persistence (mean days to discontinuation: 186; 13% discontinuation; Figs. 3 and 4).

## Discussion

Tafamidis is an innovative, first-in-class medicine to treat ATTR-CM, and the manufacturer supports an active PAP. Our study found that a high proportion of Medicare PDP patients who received tafamidis did so solely through the PAP (35%). Additionally, 14% of patients received tafamidis through both Medicare PDP and the PAP. As PAP data are not captured in the CMS Medicare PDP database, these findings imply a high risk of treatment misclassification in studies of tafamidis utilizing Medicare PDP data alone. In the absence of database cross-linkage, a patient may be misclassified as having not received tafamidis in comparative effectiveness studies using Medicare data alone, but was in fact filling the prescriptions through the PAP. As the non-treated group will include patients who received treatment, this will lead to incorrect effectiveness estimates. Further, although we found similar adherence and persistence outcomes among cohorts of patients who received treatment through only the PAP, only Medicare PDP, or both Medicare PDP and the PAP, relying solely on Medicare data underestimated mean adherence (by ~ 10%) and persistence (by ~ 2 months) in patients receiving tafamidis through both Medicare PDP and the PAP. Since innovative therapies are often costly and the manufacturers routinely offer PAPs, our study demonstrates the value of PPRL to identify a more complete treatment journey for patients accessing these treatments.

Tafamidis was approved for use in the United States in May 2019^14^ and has an ongoing PAP to support access for those with lower incomes. The identification period for the analysis of tafamidis prescriptions started at the FDA approval date and continued for 2.6 years. Although the index rate among patients who only received treatment through Medicare PDP remained stable throughout, we observed a decreasing index rate among those who received treatment through both Medicare PDP and the PAP, and an increasing index rate among those who received treatment only through the PAP. These findings were reflected in the overall follow-up durations and likely indicate an increased awareness and utilization of both tafamidis and the PAP over time. Despite minor temporal differences, index characteristics of patients who received tafamidis through these different routes were qualitatively similar, which suggests that the overall patient population changed little through 2019 to 2021, and that none of the summarized characteristics were directly related to the chosen source of treatment. As PAPs are often provided to support access to innovative therapies in patients who may otherwise have difficulties affording treatment, household income may be a factor in patient’s choice to seek access to innovative therapies through the PAP rather than Medicare PDP.

Though there were minor differences in mean persistence among the 3 route of access cohorts, these were < 40 days, and unlikely to be clinically significant. Adherence was similarly high in all cohorts, and in a range not commonly seen for other prescription medicines. Patients with heart failure show a wide variability in adherence, but rates > 85% are uncommon^15,16^. When further assessing outcomes in patients who received treatment through both Medicare PDP and the PAP, we found adherence and persistence would be underestimated if solely assessed using Medicare PDP claims. Mean adherence was underestimated by 12% by mMPR (percentage who were ≥ 80% adherent was underestimated by 25%) and 11% (percentage who were ≥ 80% adherent was underestimated by 22%) by PDC; whereas persistence was underestimated by 65 days (discontinuation overestimated by 1%) in Medicare PDP data alone. These may be clinically relevant differences that could also occur in studies of other drugs with PAPs.

The use of PPRL, in particular tokenizing technology, has great potential to allow researchers to answer new questions through anonymous linkage of multiple real-world data sources. It offers benefits to both patients and the healthcare industry through the ability to maintain data privacy and enhance security, while offering improved interoperability. The tokenization approach taken by Datavant has been validated and is compliant with the US Health Insurance Portability and Accountability Act^3,4^. Demographic PII are 1-way hashed, and the tokens are irreversible and site-specifically encrypted. Although token design can be adapted to maximize matching between databases, core tokens similar to those used in this study have previously shown a up to 99.9% precision and 95.5% recall in a US real-world population^3^. Although single-token matching can be used to identify most patients across distinct databases, increasing the number of tokens required for each match further increases accuracy. The balance between the risk of false positives and false negatives is defined by the number of tokens used, and should be tailored to the study question. In our study, 81% of patients in the PAP were matched using 8 distinct tokens, with false positives unlikely due to the number of tokens utilized. Among the 19% of patients left unmatched, 16% were matched with ≥ 1 token. This leaves a potential false negative rate of 16%; however, the inclusion of false negatives would introduce more error into our analysis than false positives, so we believe that this risk is acceptable. Privacy Preserving Record Linking technology has not been well utilized in real-world health analyses to date, which is likely due to accessibility barriers. The approach also has cost barriers, and residual ethical considerations, as organizations must consider the consent under which the data were provided; however, there are many possible applications of the technology, including use of PPRL to link clinical trial data with real-world data. The value of these applications and increasing availability of the technology is likely to increase its use over time.

Limitations to this analysis include that it specifically evaluates misclassification in the CMS Medicare PDP database. Other health insurance databases may have different extents of patient overlap, and some patients may have obtained tafamidis through other routes. As is common in claims data, there is a lack of information on the reasons for discontinuation. Further research would be needed to fully understand the routes of access to tafamidis and the reasons driving these decisions. We also acknowledge that the risk of exposure misclassification identified with tafamidis will not be the same for all therapies with PAPs, as each has a unique clinical and economic circumstance. As innovative treatments are typically high cost and commonly offer a PAP to support access, we believe that our findings may apply to many therapies in this category, and the risk of misclassification identified in this study should be considered. With respect to the tokenization process, this analysis used tokens derived from demographic PII from patients located in the United States. Each token has a different level of precision and recall, but several tokens would not be applicable in databases founded in other countries or comprising different patient identifiers, and this may affect the ability to match some subgroups of patients. Although these are considerations for use of the technology more widely, it is expected that several of these technological limitations could be overcome with the inherent flexibility of token design and variety of combinations. Additionally, the success of patient-matching is inherently linked to the validity and extent of completion of the demographic PII in each data source, and ensuring a single record is held for each patient in each database. Accuracy of adherence and persistence measures are also dependent on the completeness of the records and persistence is linked to the longevity in the databases, and of the study. Notably, though Medicare Advantage beneficiaries were included in the medication claims analyses, we did not have full access to full data on their medical claims, which may have led to an underestimation of the level of comorbidities.

In conclusion, this analysis of Medicare PDP beneficiaries, we found a high proportion of patients received tafamidis only through the PAP, or through both Medicare PDP and the PAP. This suggests a high risk of exposure misclassification in studies of tafamidis utilizing Medicare data alone. We additionally found that adherence and persistence were underestimated in patients who received tafamidis through Medicare PDP and the PAP when solely using Medicare data. These findings may also apply to other innovative therapies with PAPs, and we propose PPRL using Datavant tokens as a valuable method to allow anonymous patient-matching through multiple claims databases, allowing creation of a more complete picture of a patient’s treatment journey.

## Methods

This was a non-interventional, retrospective, cohort study of de-identified patient data from the Medicare PDP and tafamidis PAP databases, managed and stored by the CMS and Lash Group, respectively. The study was carried out in accordance with all relevant guidelines and regulations. The management of data and study materials conformed to US Health Insurance Portability and Accountability Act (HIPAA) standards. A limited dataset, which excluded patient-identifying information, was used for all analyses, as defined by the HIPAA Privacy Rule. Due to the sole use of retrospective, de-identified, and commercially available structured data, the study was granted an institutional review board waiver by Advarra (Columbia, MD, USA), and informed consent was not required.

### Data sources

Over 35 million people in the United States are beneficiaries of Medicare insurance, which primarily provides access to healthcare for patients aged ≥ 65 years but also supports younger patients with disabilities or severe chronic conditions^17^. Medicare coverage has defined ‘Parts’, with Parts A and B covering hospital inpatient and outpatient services, and Part D covering prescription drugs. Medicare Advantage plans also cover prescription drugs. People with Medicare fee-for-service Part D or Advantage coverage were classed as PDP beneficiaries in this analysis. Medicare PDP is fully paid by the CMS, which results in complete reporting of claimed and adjudicated treatment across a range of providers. The database also includes detailed demographic and general health data.

The tafamidis PAP is provided on behalf of Pfizer. It enables free treatment for patients who are unable to afford or access tafamidis and meet specific criteria. The PAP collects basic demographic, clinical characteristic, and prescription fill data from enrolled patients. During the period of this study, tafamidis was the only FDA-approved disease-modifying treatment for patients with ATTR-CM.

### Privacy preserving record linking

Privacy Preserving Record Linking tokenization technology from Datavant, Inc.^3,4,6^ was used to match patients in the CMS Medicare PDP and PAP databases. Patients with ≥ 1 tafamidis meglumine 80 mg or tafamidis free acid 61 mg prescription in the Medicare PDP or PAP database between May 3, 2019 (the date that tafamidis was approved for use in the United States^14^) and December 31, 2021, were included in the tokenization process. The matching algorithm leveraged hashed demographic PII including first name, last name, date of birth, sex, and address to create a set of unique, irreversible tokens for each patient (Fig. 1A). Each token was encrypted and formed a universal, de-identified key that can be used to reference the patient across databases while preserving privacy. Increasing the number of single-token matches required to determine that the same patient is in both databases increases the accuracy of matching, avoiding false positives. The PPRL process was compliant with the US HIPAA compliant.

### Cohort definitions

After PPRL, inclusion and exclusion criteria were applied to define 3 distinct patient cohorts: 1) those who received tafamidis through Medicare PDP only; 2) through the PAP only; or 3) through both Medicare PDP and PAP, during the time between index and end of follow-up.

Patients in all cohorts must have been aged ≥ 65 years, had Medicare FFS Parts A or B, and PDP coverage (regardless of PAP enrollment), and ≥ 2 tafamidis prescriptions at the approved dose recorded on distinct service dates across the Medicare PDP or PAP databases between May 3, 2019 and December 31, 2021. The index date for each patient was the date of first tafamidis prescription within the identification period, and patients were followed until disenrollment in Medicare PDP, death, or the end of the study on December 31, 2021.

### Cohort characterization

Demographics and clinical characteristics were summarized descriptively for the 3 cohorts. Comorbidities were summarized at index using Medical Dictionary for Regulatory Activities preferred terms, and the Chronic Conditions Data Warehouse algorithm to identify chronic conditions occurring in the same year as the patient’s index. Cardiovascular medicines taken at index were summarized using Anatomical Therapeutic Chemical codes. Data for medical claims were incomplete for people with Medicare Advantage coverage.

### Outcomes and analysis

All outcomes are descriptive. Adherence and persistence were assessed in the 3 patient cohorts and additionally in patients with ≥ 2 Medicare PDP fills and ≥ 1 PAP fill, with and without PAP data.

Adherence was assessed using both the mMPR and the PDC measures, where follow-up was considered the time between the first prescription and disenrollment in Medicare PDP, their last prescription fill, death, or the end of the study. The mMPR was calculated as the percentage of days supplied out of all days between the first and last dispensing:

$$mMPR = \frac{{\left( {\begin{array}{*{20}c} {Sum\; of \;days \;supplied\; for \;all \;refills \;over\; the\; period} \\ { - last\; days \;supply} \\ \end{array} } \right)}}{{\left( {Days \;between \;first\; and\; last \;prescription} \right)}} \times 100$$

The PDC was calculated as the % of days covered by prescription fills out of all the days between first and last prescription fill dates:

$$PDC = \frac{{\left( {Sum \;of\; days \;covered \;over \;the\; period - last\; days\; covered} \right)}}{{\left( {Days\; in \;the\; period} \right)}} \times 100$$

Adherence measures were calculated separately, with a score of ≥ 80% considered adherent in each. For the persistence calculation, patients with an index date after June 30, 2021, were excluded to allow for all patients to have at least 6 months of follow-up. An observed gap of > 60 days was considered a discontinuation for the main analysis, with a > 90 days gap explored in a sensitivity analysis shown in Supplemental Fig. 1. Follow-up was defined as the time between the first and last prescription, plus the days supplied at the last refill.

### Supplementary Information

Supplementary Information.

## Data Availability

The data that support the findings of this study are available from the Lash Group and CMS, but restrictions apply to their availability. Data were used under license for the current study and are not publicly available. For more information on data availability, please reach out to the corresponding author, Haechung Chung.
