# Supplementary material for: Adherence and persistence to tafamidis treatment among Medicare beneficiaries in the presence of a patient assistance program
Source: Sci Rep. 2024 Jul 15;14:16261. doi: 10.1038/s41598-024-62660-5 (PMC11251142; doi:10.1038/s41598-024-62660-5)
Supplement: Supplementary file 1 — Supplementary Information. [file 41598_2024_62660_MOESM1_ESM.pdf]

Supplementary Material

Adherence and persistence to tafamidis treatment among Medicare beneficiaries in the presence of a patient assistance program

Haechung Chung[1*], Cera Cantu[2], Cindi Pankratova[1], Jason Kemner[1], Jose Alvir[1], Sapna Prasad[2], Yong Chen[1]

[1]Pfizer Inc, New York, NY, USA; [2]Clarify Health Solutions. New York, NY, USA

***Corresponding author:** Haechung Chung, Pfizer Inc, New York, NY, USA.
Haechung.Chung@pfizer.com

Supplementary Table 1: Disease-relevant comorbidities in the 12 months prior to index

	Received tafamidis prescriptions during follow-up from:			
**Patients, n (%)**	**Medicare PDP only n=3877**	**PAP only n=2657**	**Both Medicare PDP and the PAP n=1020**	**All patients n=7554**
**Cardiovascular comorbidities**	2428 (63)	1611 (61)	670 (66)	4709 (62)
Amyloidosis	2152 (56)	1465 (55)	620 (61)	4237 (56)
Cardiac arrhythmias	1889 (49)	1269 (48)	527 (52)	3685 (49)
Cardiomyopathy	2037 (53)	1370 (52)	583 (57)	3990 (53)
Heart Failure	2066 (53)	1400 (53)	579 (57)	4045 (54)
Hypertension	2066 (53)	1417 (53)	585 (57)	4068 (54)
Ischemic heart disease	1600 (41)	1085 (41)	435 (43)	3120 (41)
Pacemaker or ICD	613 (16)	467 (18)	193 (19)	1273 (17)
Peripheral vascular disease	390 (10)	272 (10)	102 (10)	764 (10)
Aortic stenosis	629 (16)	461 (17)	177 (17)	1267 (17)
Diabetes	773 (20)	531 (20)	197 (19)	1501 (20)
Cerebrovascular disease	501 (13)	374 (14)	122 (12)	997 (13)
Conduction disorders	1028 (27)	712 (27)	281 (28)	2021 (27)
Pulmonary embolism	79 (2)	48 (2)	21 (2)	148 (2)
Pericarditis	300 (8)	191 (7)	64 (6)	555 (7)
Venous thrombosis	161 (4)	112 (4)	43 (4)	316 (4)
Heart transplant	27 (1)	13 (<1)	<11	*Suppressed*
**Eye disorders**	1194 (31)	764 (29)	341 (33)	2299 (30)
Cataracts	791 (20)	515 (19)	249 (24)	1555 (21)
Glaucoma	524 (14)	337 (13)	129 (13)	990 (13)
Vitreous opacities	450 (12)	273 (10)	114 (11)	837 (11)
**Genitourinary system disorders**	1234 (32)	831 (31)	332 (33)	2397 (32)
Chronic kidney disease	1119 (29)	763 (29)	303 (30)	2185 (29)
Erectile dysfunction	206 (5)	144 (5)	58 (6)	408 (5)

**Nervous system disorders**	964 (25)	618 (23)	288 (28)	1870 (25)
Peripheral neuropathy	490 (13)	343 (13)	151 (15)	984 (13)
Autonomic neuropathy	79 (2)	53 (2)	26 (3)	158 (2)
Carpal tunnel syndrome	375 (10)	223 (8)	121 (12)	719 (10)
Lumbar spinal stenosis	406 (10)	258 (10)	118 (12)	782 (10)
**Musculoskeletal and connective tissue disorders**	<11	<11	<11	12 (<1)
Atraumatic Achilles tendon rupture	<11	<11	<11	<11
Atraumatic biceps tendon rupture	<11	<11	<11	<11
**Other comorbidities**				
Monoclonal gammopathy	263 (7)	191 (7)	67 (7)	521 (7)
**No comorbidities**	1428 (37)	1034 (39)	342 (34)	2804 (37)

Low n values (<11) and those which would allow back-calculation of low values were suppressed.
Medical claims were incomplete for patients with Medicare Advantage coverage.
ICD, implantable cardioverter-defibrillator; PAP, patient assistance program; PDP, prescription drug plan.

Supplementary Table 2: Chronic conditions from the Chronic Conditions Data Warehouse algorithm

	Received tafamidis prescriptions during follow-up from:			
Patients, n (%)	Medicare PDP only n=3877	PAP only n=2657	Both Medicare PDP and the PAP n=1020	All patients n=7554
Acute myocardial infarction	159 (4)	109 (4)	33 (3)	301 (4)
Alzheimer's disease	40 (1)	20 (1)	<11	*Suppressed*
Anemia	1009 (26)	701 (26)	242 (24)	1952 (26)
Asthma	307 (8)	169 (6)	73 (7)	549 (7)
Atrial fibrillation and flutter	1599 (41)	1081 (41)	464 (45)	3144 (42)
Benign prostatic hyperplasia	915 (24)	609 (23)	239 (23)	1763 (23)
Breast cancer	43 (1)	39 (1)	12 (1)	94 (1)
Cataract	706 (18)	474 (18)	225 (22)	1405 (19)
Chronic kidney disease	1184 (31)	819 (31)	329 (32)	2332 (31)
Chronic obstructive pulmonary disease	309 (8)	233 (9)	66 (6)	608 (8)
Colorectal cancer	47 (1)	37 (1)	18 (2)	102 (1)
Depression, bipolar, or other depressive mood disorders	366 (9)	276 (10)	107 (10)	749 (10)
Diabetes	745 (19)	508 (19)	195 (19)	1448 (19)
Endometrial cancer	<11	<11	<11	<11
Glaucoma	512 (13)	347 (13)	137 (13)	996 (13)
Heart failure and non-ischemic heart disease	2254 (58)	1507 (57)	628 (62)	4389 (58)
Hip/pelvic fracture	22 (1)	21 (1)	<11	*Suppressed*
Hyperlipidemia	1946 (50)	1359 (51)	538 (53)	3843 (51)
Hypertension	2077 (54)	1412 (53)	581 (57)	4070 (54)
Hypothyroidism	547 (14)	364 (14)	152 (15)	1063 (14)
Ischemic heart disease	1425 (37)	987 (37)	396 (39)	2808 (37)
Lung cancer	35 (1)	21 (1)	<11	*Suppressed*

**Non-Alzheimer's dementia**	172 (4)	89 (3)	32 (3)	293 (4)
**Osteoporosis with or without pathological fracture**	162 (4)	127 (5)	38 (4)	327 (4)
**Parkinson's disease and secondary Parkinsonism**	43 (1)	28 (1)	18 (2)	89 (1)
**Pneumonia, all-cause**	255 (7)	181 (7)	67 (7)	503 (7)
**Prostate cancer**	328 (8)	239 (9)	108 (11)	675 (9)
**Rheumatoid arthritis/ osteoarthritis**	1307 (34)	878 (33)	355 (35)	2540 (34)
**Stroke/transient ischemic attack**	298 (8)	161 (6)	65 (6)	524 (7)
**Urologic cancer**	35 (1)	31 (1)	13 (1)	79 (1)

Low n values (<11) and those which would allow back-calculation of low values were suppressed.
Medical claims were incomplete for patients with Medicare Advantage coverage.
PAP, patient assistance program; PDP, prescription drug plan.

Supplementary Table 3: Cardiovascular medications taken at index

	Received tafamidis prescriptions during follow-up from:			
**Patients, n (%)**	**Medicare PDP only n=3877**	**PAP only n=2657**	**Both Medicare PDP and the PAP n=1020**	**All patients n=7554**
**Angiotensin-converting-enzyme inhibitors**	982 (25)	689 (26)	272 (27)	1943 (26)
**Angiotensin receptor blockers**	985 (25)	698 (26)	252 (25)	1935 (26)
**Angiotensin receptor-neprilysin inhibitors**	294 (8)	246 (9)	67 (7)	607 (8)
**Aldosterone antagonists**	1368 (35)	973 (37)	373 (37)	2714 (36)
**Beta-blockers**	2667 (69)	1908 (72)	719 (70)	5294 (70)
**Calcium channel blockers**	929 (24)	691 (26)	245 (24)	1865 (25)
**Diuretics**	3391 (87)	2336 (88)	886 (87)	6613 (88)
**Any anticoagulants**	2409 (62)	1589 (60)	635 (62)	4633 (61)
Warfarin	386 (10)	368 (14)	134 (13)	888 (12)
Direct oral anticoagulants	2120 (55)	1293 (49)	521 (51)	3934 (52)
**Any antiplatelet medications**	419 (11)	329 (12)	105 (10)	853 (11)
P2Y12 inhibitors	414 (11)	329 (12)	104 (10)	847 (11)
Aspirin	<11	<11	<11	<11
**No medications**	118 (3)	78 (3)	34 (3)	230 (3)

Low n values (<11) were suppressed.
PAP, patient assistance program; PDP, prescription drug plan.

Supplemental Figure 1: Persistence (sensitivity analysis with 90-day gap)

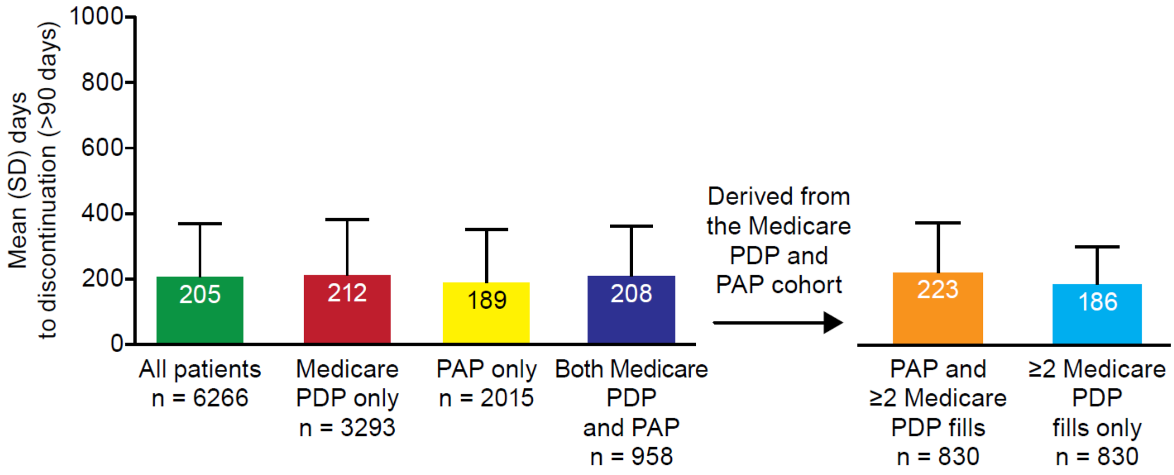

	All patients	Medicare PDP only	PAP only	Both Medicare PDP and PAP	PAP and ≥2 Medicare PDP fills	≥2 Medicare PDP fills only
Patients who discontinued	4%	4%	3%	5%	5%	11%
Median (Q1, Q3)	170 (59, 330)	177 (66, 330)	136 (30, 328)	171 (77, 345)	189 (97, 356)	173 (104, 252)

PAP, patient assistance programs; PDP, prescription drug plan; Q, quartile; SD, standard deviation
